# Suspected primary hyperreninism in a cat with malignant renal sarcoma and global renin‐angiotensin‐aldosterone system upregulation

**DOI:** 10.1111/jvim.16329

**Published:** 2021-12-03

**Authors:** Jeremy Evans, Jessica Ward, Oliver Domenig, Jonathan P. Mochel, Kate Creevy

**Affiliations:** ^1^ Department of Small Animal Clinical Sciences Texas A&M College of Veterinary Medicine & Biomedical Sciences College Station Texas USA; ^2^ Veterinary Clinical Sciences Iowa State University College of Veterinary Medicine Ames Iowa USA; ^3^ Attoquant Diagnostics Vienna Austria; ^4^ Department of Biomedical Sciences, SMART Pharmacology Iowa State University Ames Iowa USA

**Keywords:** chronic kidney disease, endocrinopathy, hyperaldosteronism, hypertension, reninoma

## Abstract

A 14‐year‐old male castrated domestic medium‐hair cat with diabetes mellitus was evaluated for vomiting, diarrhea, and anorexia. Two weeks before presentation, the cat had been diagnosed with congestive heart failure and started on furosemide. Initial diagnostic testing identified hypokalemia, systemic hypertension, and hypertrophic cardiomyopathy phenotype, and plasma aldosterone concentration was moderately increased. Abdominal ultrasound examination disclosed bilateral adrenomegaly and a right renal mass, and cytology of a needle aspirate of the mass was consistent with malignant neoplasia. The cat was treated with amlodipine and spironolactone. Because of the unusual presentation for hyperaldosteronism, a comprehensive profile of renin‐angiotensin‐aldosterone system (RAAS) peptides was performed. Results from multiple timepoints indicated persistently and markedly increased plasma renin activity and generalized RAAS upregulation. In addition to the lack of adrenal tumor, the markedly increased plasma renin activity was atypical for primary hyperaldosteronism. These clinical findings are suggestive of primary hyperreninism, a condition previously unreported in cats. The concurrent presence of a renal neoplasm suggests the possibility of a renin‐secreting tumor.

**Abbreviations**
ACE: angiotensin‐converting enzyme
AngI: angiotensin I
AngII: angiotensin II
AngIII: angiotensin III
AngIV: angiotensin IV
AP: aminopeptide
BUN: blood urea nitrogen
CKD: chronic kidney disease
PA: primary hyperaldosteronism
PRA: plasma renin activity
RAAS: renin‐angiotensin‐aldosterone system
RI: reference interval

## 1 INTRODUCTION

The renin‐angiotensin‐aldosterone system (RAAS) is a complex hormonal system that regulates intravascular volume, serum electrolyte concentrations, and renal perfusion. The release of renin from renal juxtaglomerular cells, stimulated by decreases in renal blood flow and salt delivery to distal tubules, initiates this system by the conversion of angiotensinogen (produced by the liver) to angiotensin I (AngI). Angiotensin I is converted to angiotensin II (AngII) by angiotensin‐converting enzyme (ACE), and AngII has potent systemic affects including vasoconstriction, increased intraglomerular blood pressure, renal retention of sodium and water, and AngII also stimulates the release of antidiuretic hormone and aldosterone. These downstream hormones further amplify the effects of AngII and lead to an overall increase in systemic blood pressure and glomerular filtration rate.^1^

The activity of the RAAS is related to both renal and cardiovascular status. Although the RAAS is intended to be protective against hypovolemia or hypotension, abnormal activation of this system can be seen with disease processes such as chronic kidney disease (CKD) and cardiac disease. In these instances, excessive RAAS activation can further exacerbate the underlying disease through mechanisms including systemic hypertension, glomerulosclerosis, and myocardial fibrosis.^2^, ^3^, ^4^, ^5^ For this reason, RAAS‐inhibiting agents, such as ACE or renin inhibitors, frequently are utilized as adjunctive treatments.^1^

Beyond the traditional pathway of renin, AngI, ACE, and AngII, recent research has identified additional components of the RAAS. The discovery of ACE2, as well as multiple alternative downstream angiotensin peptides including angiotensin III (AngIII), angiotensin IV (AngIV), Ang(1‐7), and Ang(1‐5), provides further insight into the complex nature of this system as well as offering potential targets for future research and treatments.^6^, ^7^ Interestingly, some of these novel peptides have been shown to have positive systemic effects and antagonize the action of AngII, which may depend not only on the specific peptide, but also on its concentration and the type of receptor that is bound.^7^ Some RAAS‐inhibiting agents, such as telmisartan, may exert beneficial affects not only by impeding the traditional RAAS, but also by diverting RAAS metabolites to these alternative beneficial pathways.^7^, ^8^

In cats, the relative concentrations of such new peptides (as well as possible derangements in the presence of systemic disease) are not well established. Consideration of both classical and alternative RAAS pathways is crucial for the understanding and management of disease. Comprehensive RAAS profiling has been performed in healthy cats, as well as in cats with cardiomyopathy^8^, ^9^ or systemic hypertension.^9^ Results of these studies suggest that among cats with cardiomyopathy, RAAS profiles remain unchanged compared to healthy cats during the early and asymptomatic phase.^8^ In contrast, cats with more advanced disease and congestive heart failure show significant and nonspecific RAAS upregulation, with the most marked RAAS perturbations occurring in furosemide‐treated cats.^9^ Among cats with systemic hypertension, untreated cats did not differ from healthy controls, whereas significant nonspecific RAAS upregulation was observed in the subset of cats receiving amlodipine.^9^ In this case report, we describe the use of a comprehensive RAAS profile to document markedly increased concentrations of both classical and alternative RAAS aminopeptides (APs) in a cat with hyperaldosteronism, systemic hypertension, congestive heart failure, CKD, and a renal mass, leading to suspicion of hyperreninism secondary to a renin‐secreting tumor.

## 2 CASE DESCRIPTION

A 14‐year‐old 8 kg male castrated domestic medium‐hair cat was evaluated at the Texas A&M University Veterinary Medical Teaching Hospital for chronic vomiting and new onset of diarrhea and anorexia. Five years before presentation, the cat had been diagnosed with diabetes mellitus and was receiving insulin glargine (6 units [0.75 U/kg] SC q12h). Clinicopathologic findings 5 months before this presentation were consistent with International Renal Interest Society (IRIS) stage 2 CKD. Two weeks before presentation, the cat was evaluated for respiratory distress at a local clinic and a presumptive diagnosis of congestive heart failure was made. The cat was discharged on furosemide (1.5 mg/kg PO q12h) and a decreased insulin dose to accommodate decreased appetite (4 units [0.5 U/kg] SC q12h). The cat's respiratory signs reportedly resolved with furosemide treatment.

Upon presentation, the cat was eupneic with a respiratory rate of 28 breaths per minute and a normal heart rate. A gallop sound was auscultated, and a grade III/VI parasternal murmur was heard in both systole and diastole. The patient was quiet but responsive, estimated to be approximately 7% dehydrated, and was considered to be obese with a body condition score of 8/9. Venous blood analysis identified moderate hypoglycemia (49 mg/dL; reference interval [RI], 83‐112), hypokalemia (2.83 mmol/L; RI, 3.91‐4.4), and mild azotemia (serum creatinine concentration, 2.5 mg/dL; RI, 0.2‐2.5; blood urea nitrogen [BUN] concentration, 43 mg/dL; RI, 7‐32). A CBC identified leukocytosis (19.93 × 10^3^/μL; RI, 2.87‐17.02) characterized by mature neutrophilia (17.34 × 10^3^/μL; RI, 2.30‐10.29) and no other abnormalities. A serum biochemistry panel confirmed persistent hypoglycemia (30 mg/dL; RI, 71‐159), hypokalemia (2.5 mmol/L; RI, 3.5‐5.8), and azotemia (serum creatinine concentration, 3.0 mg/dL; RI, 0.8‐2.4; BUN concentration, 43 mg/dL; RI, 16‐36). Mild hyperglobulinemia also was identified (5.3 g/dL; RI, 2.8‐5.1). Urinalysis disclosed a urine specific gravity of 1.016 with glucosuria (50 mg/dL) with no ketones or protein detected. Serum fructosamine concentration was consistent with prolonged or recurrent hypoglycemia (252 μmol/L; RI, <350 for nondiabetic cats). Thoracic and abdominal radiographs identified an enlarged cardiac silhouette, small and irregular kidneys suggestive of bilateral CKD, and nephroliths in the right kidney. A point‐of‐care N‐terminal pro‐B‐type natriuretic peptide (SNAP Feline proBNP Test; IDEXX Laboratories, Inc, Westbrook, Maine) result was abnormal. Average Doppler blood pressures were consistent with systemic hypertension (172 ± 14 mm Hg). The cat was hospitalized and received conservative IV fluid support (45 mL/kg/d lactated Ringer's solution) with potassium (KCl) and dextrose supplementation, as well as the previously prescribed PO furosemide. Insulin was discontinued until the patient was no longer hypoglycemic and eating voluntarily.

The next day, the cat's appetite returned and repeat blood testing identified hyperglycemia (306 mg/dL; RI, 65‐131) with persistent but improved hypokalemia (3.1 μmol/L; RI, 3.5‐5.1) and azotemia (serum creatinine concentration, 2.36 mg/dL; RI, 0.8‐1.8; BUN concentration, 43 mg/dL; RI, 19‐33). Insulin was restarted at 1 unit (0.125 U/kg) SC q12h and blood glucose concentration was monitored regularly q2‐4h. The cat ultimately was discharged on 1 unit insulin glargine SC q24h.

**FIGURE 1:**
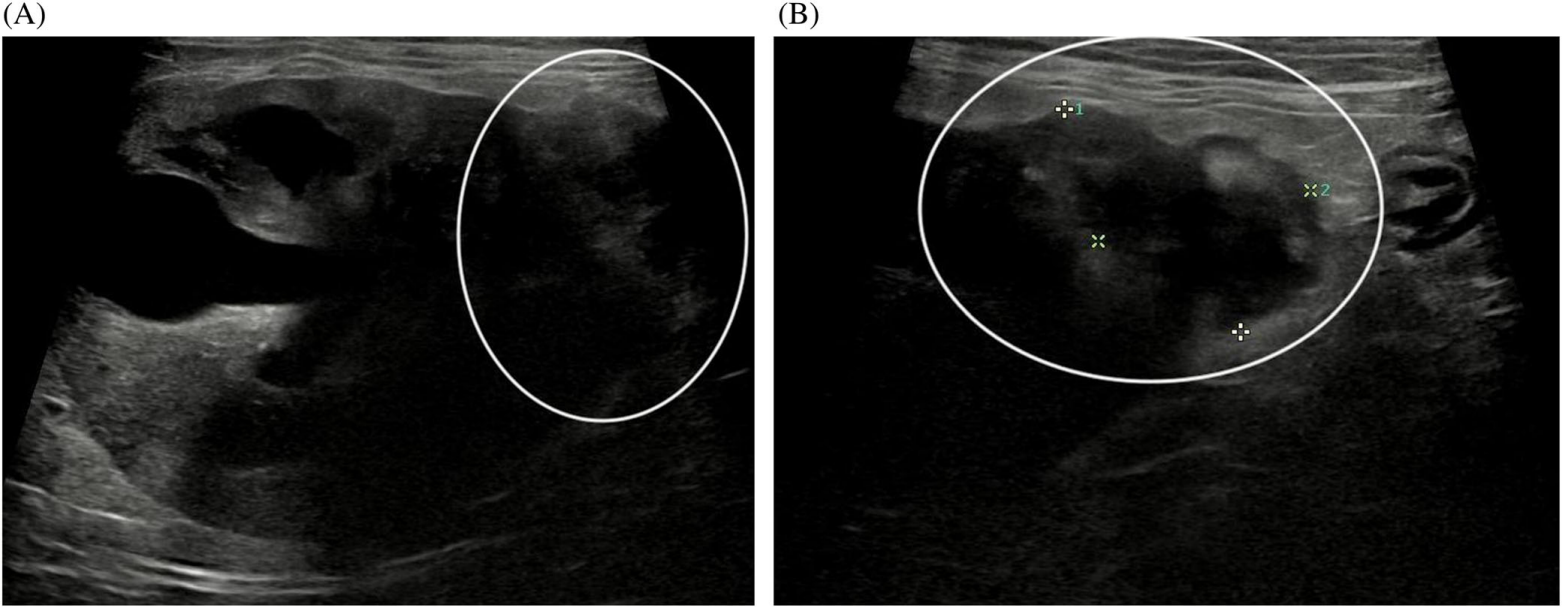
The patient's right renal mass (A, B). On abdominal ultrasound, a heterogenous mass was identified at the caudal pole of the right kidney (white circle)

**FIGURE 2:**
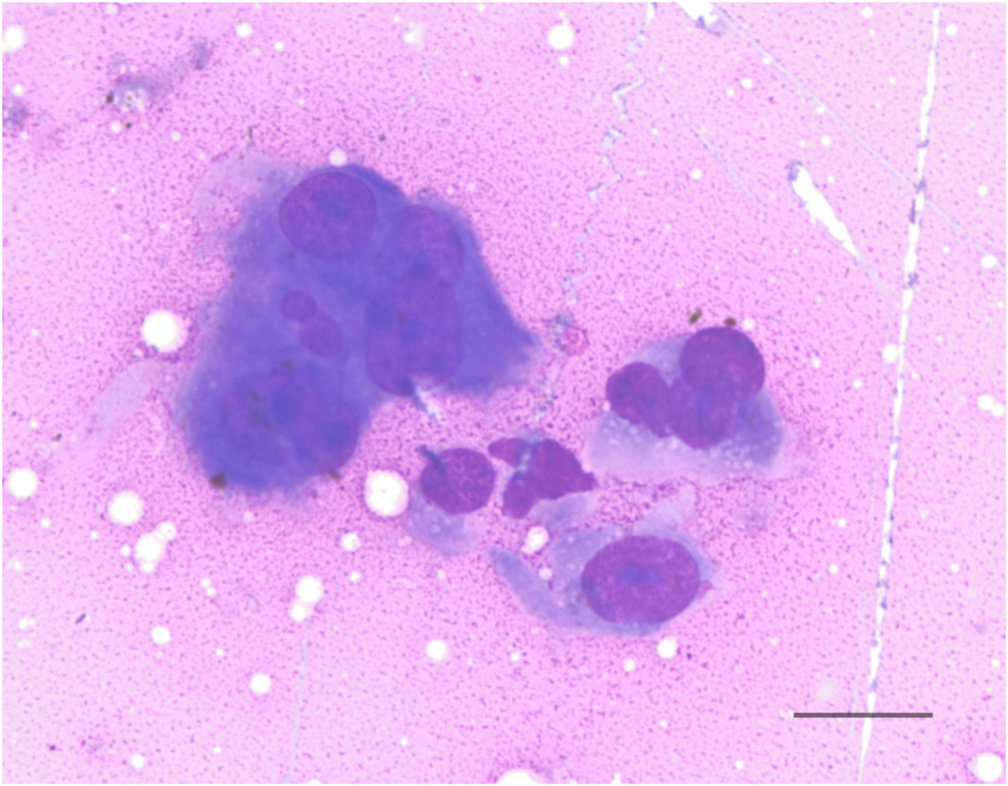
Cytology of a fine‐needle aspirate of the cat's renal mass (Wright's stain, ×60 magnification). Cells displayed atypia with anisokaryosis, prominent nucleoli, and nuclear molding. Cytology was consistent with a malignant neoplasia with a sarcoma considered most likely. Scale bar = 20 μm

An echocardiogram identified symmetrical left ventricular concentric hypertrophy, severe left atrial enlargement, moderate mitral valve regurgitation, and moderate aortic insufficiency. These findings were consistent with hypertrophic cardiomyopathy phenotype with systemic hypertension, and severity of disease was consistent with the previously suspected congestive heart failure. The patient subsequently was started on amlodipine (0.625 mg PO q24h), clopidogrel (18.75 mg PO q24h), and a decreased furosemide dose (0.75 mg/kg PO q12h) because of azotemia. The patient's thyroid status was normal (serum total thyroxine concentration, 1.94 μg/dL; RI, 0.78‐3.82). Abdominal ultrasound examination identified bilateral chronic degenerative renal changes, bilateral moderate adrenomegaly (left, 0.6 × 1.8 cm; right, 0.7 × 1.8 cm) and a 2.1 × 1.6 cm heterogeneous, predominantly hypoechoic, mass at the caudal pole of the right kidney (Figure 1). A fine needle aspirate of the renal mass was evaluated by a board‐certified clinical pathologist and diagnosed as malignant neoplasia with cytologic features most consistent with sarcoma, but an anaplastic carcinoma also was considered possible (Figure 2). Based on these diagnostic test results, the cat was diagnosed with hypertrophic cardiomyopathy phenotype with hypertension and historical congestive heart failure, diabetes mellitus, a malignant renal neoplasm of unclear clinical relevance, and possible hyperaldosteronism (with resultant hypokalemia and systemic hypertension). A serum aldosterone concentration had been submitted (Aldosterone, baseline; Michigan State University Veterinary Diagnostic Laboratory, Lansing, Michigan), but results were not available at the time of discharge. The cat was discharged on furosemide, amlodipine, clopidogrel, insulin glargine, and potassium gluconate supplementation.

Three weeks after discharge, the cat was doing well at home and a serum fructosamine measurement was consistent with appropriate diabetic management. The patient remained hypertensive (170‐180 mm Hg) and hypokalemic (3.0 mmol/L), and the previously submitted serum aldosterone concentration was increased (785 pmol/L; RI, 194‐388). This test result supported the clinical diagnosis of hyperaldosteronism and prompted the addition of spironolactone (0.75 mg/kg PO q12h). The amlodipine dose was not increased at this time because of concern for additive effects on blood pressure. The patient again was evaluated 8 weeks after discharge for recurrence of lethargy, hyporexia, and substantial weight loss. Systolic blood pressure, serum fructosamine concentration, and a repeat total T4 concentration were within normal limits. A serum biochemistry profile disclosed static azotemia and hypokalemia as well as hyperglobulinemia (4.6 g/dL; RI, 2.3‐3.8). Further imaging diagnostics (repeat abdominal ultrasound, computed tomography) were declined by the owner.

Because of the cat's unusual hyperaldosteronism phenotype involving bilateral adrenomegaly (rather than a discrete adrenal mass), as well as the presence of a presumptive malignant renal mass, an AP profile (RAS‐Fingerprint, Attoquant Diagnostics, Vienna, Austria) was performed to investigate whether components of the RAAS in addition to aldosterone were dysregulated. Repeated venous blood samples were collected on a single day, 1, 4, and 9 hours after receiving all current medications that morning (furosemide, amlodipine, clopidogrel, spironolactone, and potassium gluconate). Insulin was the only medication administered during the course of sample collection. Within a month after blood sampling for RAAS quantification, the cat's lethargy and weight loss progressed, prompting the owners to elect euthanasia. Permission for necropsy was declined.

## 3 COMPLETE RAAS PROFILE

The plasma equilibrium concentrations of 6 different RAAS angiotensin peptides, including AngI, AngII, AngIII, AngIV, Ang(1‐7), and Ang(1‐5) were quantified by liquid chromatography‐mass spectrometry/mass spectroscopy performed at a commercial laboratory using previously validated and described methods.^8^, ^10^ Angiotensin‐based markers for renin (PRA‐S) and angiotensin converting enzyme (ACE‐S) were derived from AngII and AngI concentrations by calculating their sum and ratio, respectively, as described by the analytical laboratory.^11^, ^12^

The results obtained were compared to RAAS profile results obtained from 34 healthy cats, 17 cats with underlying cardiomyopathy (7 of which received furosemide), and 15 cats with systemic hypertension (7 of which received amlodipine).^9^ As shown in Tables 1 and 2, concentrations of RAAS angiotensin peptides in our cat were markedly increased compared to those of healthy cats, and in most cases exceeded the 75% percentile for cats with cardiomyopathy or systemic hypertension. Table 3 includes only treated cats from each disease group (amlodipine for systemic hypertension and furosemide for congestive heart failure). In this comparison, our cat's results did not frequently exceed the 75th percentile, but the calculated PRA and AngII concentration still exceeded those of furosemide‐treated cats with cardiomyopathy. Increases in RAAS metabolites were persistent across timepoints, with minimal variation based on time of sampling with respect to drug administration.

**TABLE 1:** Plasma equilibrium concentrations of RAAS aminopeptides in a cat with suspected hyperreninism compared to healthy cats, cats with cardiomyopathy, and cats with systemic hypertension

	RAAS biomarker	AngI (pmol/L)	AngII (pmol/L)	Aldosterone (pmol/L)	AngIII (pmol/L)	AngIV (pmol/L)	Ang1‐7 (pmol/L)	Ang1‐5 (pmol/L)
Patient	1‐h post	1252.5	860.9	724.1	23.7	16.7	425.7	360.6
	4‐h post	1073.7	842.8	594.6	26.6	13.3	410.5	443.3
	9‐h post	1424.1	990.1	564.8	24.4	14.5	505.1	461.4
Group data, median (IQR)	Healthy cats	37.8 (22.5‐60.1)	122.6 (76.0‐211.6)	148.4 (102.0‐182.8)	7.48 (2.50‐13.44)	2.95 (2.00‐6.15)	19.9 (13.5‐37.2)	136.1 (54.6‐224.3)
	Cardiomyopathy	178.5 (89.9‐409.3)	159.7 (126.7‐725.3)	318.6 (240.3‐700.2)	9.40 (4.40‐19.80)	4.31 (2.80‐19.10)	109.6 (48.7‐170.6)	466.2 (73.4‐668.3)
	Systemic hypertension	103.2 (28.2‐513.5)	188.9 (49.9‐322.6)	239.6 (138.1‐373.6)	10.30 (2.50‐25.55)	6.60 (2.00‐12.50)	65.8 (18.5‐225.7)	119.1 (51.9‐260.3)

Abbreviations: AngI, angiotensin I; AngII, angiotensin II; AngIII, angiotensin III; AngIV, angiotensin IV; IQR, interquartile range; RAAS, renin‐angiotensin‐aldosterone system.

**TABLE 2:** Calculated plasma renin and angiotensin‐converting enzyme activities (PRA‐S and ACE‐S, respectively) in a cat with suspected hyperreninism compared to healthy cats, cats with cardiomyopathy, and cats with systemic hypertension

		PRA‐S (pmol/L)	ACE‐S ([pmol/L]/[pmol/L])
Patient	1‐h post	2113.5	0.69
	4‐h post	1916.4	0.78
	9‐h post	2414.2	0.70
Group data, median (IQR)	Healthy cats	156.8 (106.2‐286.4)	3.35 (2.68‐3.76)
	Cardiomyopathy	325.2 (220.9‐997.1)	1.21 (0.96‐1.87)
	Systemic hypertension	318.4 (76.9‐1049.7)	1.18 (0.62‐2.92)

Abbreviation: IQR, interquartile range.

**TABLE 3:** Plasma equilibrium concentrations of RAAS aminopeptides, calculated PRA‐S, and ACE‐S from the reported case (average of 3 timepoints) and hypertensive cats receiving amlodipine and cats with cardiomyopathy receiving furosemide

RAAS biomarker	CM + furosemide (n = 7)	SHT + amlodipine (n = 7)	Patient
AngI (pmol/L)	533.5 (81.2‐826.6)	522 (436.6‐2686.6)	1250.1 (1073.7‐1424.1)
AngII (pmol/L)	725.3 (139.7‐900.9)	382.3 (220.2‐478.9)	897.9 (842.8‐990.1)
Aldosterone (pmol/L)	687.3 (264.8‐1287.3)	430.2 (261.7‐495.2)	627.9 (564.8‐724.1)
AngIII (pmol/L)	19.8 (7.1‐41.9)	34.7 (12.1‐38.05)	24.9 (23.7‐26.6)
AngIV (pmol/L)	19.1 (3.4‐32.4)	14.0 (9.95‐22.55)	14.8 (13.3‐16.7)
Ang1‐7 (pmol/L)	343.7 (31.2‐494.9)	226.6 (220.2‐831.5)	447.1 (410.5‐505.1)
Ang1‐5 (pmol/L)	668.3 (76.6‐1264.9)	94.3 (63.8‐266.4)	421.8 (360.6‐461.4)
PRA‐S (pmol/L)	1261.7 (220.9‐1727.4)	1059 (870.1‐3019.8)	2148 (1916.4‐2414.2)
ACE‐S ([pmol/L]/[pmol/L])	1.31 (1.09‐1.72)	0.34 (0.185‐1.05)	0.7 (0.69‐0.78)

Abbreviations: CM, cardiomyopathy; RAAS, renin‐angiotensin‐aldosterone system; SHT, systemic hypertension.

## 4 DISCUSSION

Our cat's clinical presentation as well as results of the RAAS peptide profile suggests a hyperreninemic state. Although some clinical abnormalities in this case, including systemic hypertension and hypokalemia, can be attributable specifically to hyperaldosteronism, the far more common cause of primary hyperaldosteronism (PA) in cats is unilateral adrenal neoplasia as opposed to the finding of bilateral adrenomegaly in this case.^13^, ^14^ Additionally, in cases of PA, the increased aldosterone concentration has an inhibitory effect on renin release, resulting in a decreased PRA.^14^ The substantial increase in PRA observed in our case is more consistent with aberrant renin production leading to generalized RAAS upregulation and secondary hyperaldosteronism.

In humans, PRA often is increased secondary to CKD, and renin‐inhibiting agents occasionally are used to manage disease sequelae.^15^ By contrast, the available literature in cats with CKD is less clear. Although earlier studies consistently document secondary hyperaldosteronism, PRA has been found to be low, normal, or high in cats with CKD.^16^, ^17^ The presence of concurrent hypertension, as seen in our cat, can decrease PRA in both normal and azotemic cats and can be associated with increased plasma aldosterone concentrations.^17^ Plasma renin activity is increased in humans with congested heart failure (CHF),^3^ and RAAS peptide profiles in dogs and cats with clinically relevant cardiac disease show RAAS upregulation.^9^, ^18^, ^19^, ^20^ In the case reported here, the echocardiographic phenotype was most consistent with secondary effects of systemic hypertension rather than primary cardiomyopathy, suggesting that hyperreninism likely precipitated the development of congestive heart failure, and not the opposite. Furthermore, extent of increase for certain RAAS components in this cat, specifically PRA and AngI, was more marked than results previously established in cats with CHF, with or without furosemide treatment.

Interpretation of the RAAS panel is complicated by the medications administered, but we attempted to limit their potential influence by collecting multiple samples throughout the course of the day after medication administration. Although no data in cats are available regarding the effect of spironolactone on RAAS components, amlodipine administration has been shown to significantly increase PRA.^9^, ^17^, ^21^ Another study found that despite having increased aldosterone concentrations at baseline, hypertensive cats receiving amlodipine did not have further increases in aldosterone concentration.^17^ In humans, most diuretics, including spironolactone and furosemide, lead to an increase in PRA, but in some patients PRA remains unchanged.^22^, ^23^, ^24^ Preliminary data suggest that furosemide may further exacerbate RAAS disturbances in cats with cardiomyopathy.^9^ Oral potassium supplementation also may increase aldosterone and PRA.^25^ Although it is recognized that the cat's medications likely altered its RAAS profile, it is considered highly unlikely that these medications solely led to such marked global dysregulation.

The presence of a malignant renal neoplasm offers a possible etiology for the suspected hyperreninism in our case. In humans, hyperreninism has been associated with renin‐producing juxtaglomerular cell tumors (reninomas), and affected patients may present with hypertensive cardiomyopathy and congestive heart failure.^26^, ^27^ Although in the present case, histopathology of the renal tumor was unavailable, a reninoma would be expected to result in similar RAAS perturbations. The persistently increased RAAS activity, independent of the timing of medication administration, also is suggestive of aberrant and autonomous renin production.

Our case report had some limitations. As previously discussed, the concurrent administration of medications that can affect RAAS status likely influenced the results obtained in this case. However, it would be difficult to characterize the RAAS of a hyperreninemic cat without the influence of such medications, because the rarity of this disease entity, combined with the severe systemic sequelae, most often would result in the initiation of such treatment before clinical consideration and subsequent testing. Necropsy and subsequent histopathology would have been beneficial to investigate the presence of a renin‐producing tumor. Additionally, follow‐up imaging, such as ultrasound or computed tomography (CT), would have provided valuable information regarding the growth and possible metastasis of the renal mass.

Regardless of the underlying etiology, the RAAS peptide profile in this cat allowed extensive characterization of its hyperreninemic state. The results of the cat's RAAS profile also provided therapeutic targets for treating its underlying hypertension and illustrated that RAAS‐modulating agents such as telmisartan or spironolactone might have been a more appropriate initial treatment than amlodipine. More in‐depth investigations of RAAS derangements in cats with CKD, hypertension, or both may further facilitate understanding the pathophysiology as well as aid clinical decision‐making.

## CONFLICT OF INTEREST DECLARATION

Dr Oliver Domenig currently works as the laboratory head of Attoquant Diagnostics in Vienna, Austria. Drs Jonathan P. Mochel and Jessica Ward have received consulting fees and research grants from Ceva Sante Animale. No other authors have a conflict of interest.

## OFF‐LABEL ANTIMICROBIAL DECLARATION

Authors declare no off‐label use of antimicrobials.

## INSTITUTIONAL ANIMAL CARE AND USE COMMITTEE (IACUC) OR OTHER APPROVAL DECLARATION

Authors declare no IACUC or other approval was needed.

## HUMAN ETHICS APPROVAL DECLARATION

Authors declare human ethics approval was not needed for this study.
